# *N*-Glycosylation Site Analysis of Citrullinated Antigen-Specific B-Cell Receptors Indicates Alternative Selection Pathways During Autoreactive B-Cell Development

**DOI:** 10.3389/fimmu.2019.02092

**Published:** 2019-09-04

**Authors:** Rochelle D. Vergroesen, Linda M. Slot, Barbera D. C. van Schaik, Marvyn T. Koning, Theo Rispens, Antoine H. C. van Kampen, René E. M. Toes, Hans U. Scherer

**Affiliations:** ^1^Department of Rheumatology, Leiden University Medical Center, Leiden, Netherlands; ^2^Bioinformatics Laboratory, Amsterdam Public Health Research Institute, Amsterdam Infection & Immunity Institute, Amsterdam UMC, University of Amsterdam, Amsterdam, Netherlands; ^3^Department of Hematology, Leiden University Medical Center, Leiden, Netherlands; ^4^Department of Immunopathology, Sanquin Research and Landsteiner Laboratory, Academic Medical Center, Amsterdam, Netherlands; ^5^Biosystems Data Analysis, Swammerdam Institute for Life Sciences, University of Amsterdam, Amsterdam, Netherlands

**Keywords:** anti-citrullinated protein antibodies, B cells, glycosylation, variable domain (Fab), rheumatoid arthritis, germinal center

## Abstract

Many autoimmune diseases are hallmarked by autoreactive B and plasma cell responses that are directly or indirectly involved in disease pathogenesis. These B-cell responses show large variability between diseases, both in terms of the secreted autoantibody repertoire and the dynamics and characteristics of the underlying B-cell responses. Hence, different mechanisms have been proposed to explain the emergence of autoreactive B cells in an otherwise self-tolerant immune system. Notably, most mechanistic insights have been obtained from murine studies using models harboring genetic modifications of B and T cells. Given recent technological advances that have rendered autoreactive human B cells accessible for analysis, we here discuss the phenomenon of extensive *N*-glycosylation of the B-cell receptor (BCR) variable domain of a prototypic human autoreactive B-cell response and its potential role in the generation of autoimmunity. Anti-citrullinated protein antibodies (ACPA) hallmark the most disease-specific autoimmune response in Rheumatoid Arthritis (RA). Remarkably, ACPA-IgG are heavily *N*-glycosylated in the variable domain due to somatic mutations that generate abundant *N*-glycosylation consensus sequences. These sites, obtained from full-length BCR sequences of ACPA-expressing B cells from 12 ACPA-positive RA patients, were here analyzed in detail. Sites that required a single nucleotide mutation to be generated were defined as single somatic hypermutation (s-SHM) sites, whereas sites requiring multiple mutations were defined as m-SHM sites. IgG sequences of 12 healthy donors were used as control. Computational modeling of the germinal center reaction (CLONE algorithm) was used with the germline counterparts of ACPA-IgG heavy chain (HC) sequences to simulate the germinal center response. Our analyses revealed an abundance of *N*-glycosylation sites in ACPA-IgG HC that frequently required multiple mutations and predominated in specific positions. Based on these data, and taking into account recent insights into the dynamics of the ACPA-response during disease development, we here discuss the hypothesis that *N*-glycosylation sites in ACPA-IgG variable domains could lead to alternative, possibly antibody affinity-independent selection forces. Presumably, this occurs during germinal center responses allowing these B cells to escape from putative tolerance checkpoints, thereby driving autoreactive B cell development in the pathogenesis of RA.

## Introduction

The mechanisms leading to the development of human B-cell mediated autoimmunity are incompletely understood. In particular, it is unclear how autoreactive B cells arise and escape tolerance checkpoints that normally control their development and expansion. While transient, short-lived autoreactive B-cell responses are frequently observed in the context of external, innate triggers such as infections, the development of long-lived, autoreactive B and plasma cells in the context of autoimmune disease likely requires T-cell help and the involvement of germinal centers (GCs) in lymph nodes or GC-like structures in inflamed tissues. As these specialized structures are equipped with various control mechanisms to prevent the development of autoreactivity (1, 2), it is important to understand why and how these protective mechanisms fail in the development of human autoimmunity. Using sequencing data of full-length variable regions of B-cell receptors (BCRs) specific for the most relevant autoantigen in rheumatoid arthritis, a prototypic autoimmune disease, we here discuss the hypothesis that human autoreactive B cells can circumvent classical mechanisms of negative selection during GC reactions by introducing *N*-glycosylation sites in the antibody variable (V-) domain. In fact, we provide evidence for the notion that the remarkable V-domain *N*-glycosylation observed for antibodies against citrullinated protein antigens (ACPA) in this disease allows for the outgrowth of autoreactive B cells that show extensive somatic hypermutation (SHM) with limited concurrent affinity maturation. Hence, the classical process of affinity maturation as a means to increase antigen specificity and to avoid negative selection is uncoupled from the process of SHM in this particular response. While the exact mechanisms by which V-domain *N*-glycans allow these B cells to escape tolerance control remain to be determined, our data provide an example of BCR diversification through abundant *N*-glycosylation sites in the antigen-specific BCR repertoire of a human autoreactive B-cell response and form the basis for further exploration of this intriguing phenomenon.

## Rheumatoid Arthritis

Rheumatoid arthritis is a chronic inflammatory disease that affects ~1% of the population (3). The majority of patients harbors autoantibodies, of which those most frequently observed target protein antigens in which arginine residues have undergone enzymatic, posttranslational modification into the non-classical amino acid citrulline (4). Citrulline is the common determinant recognized by the antibodies generated. In principal, any arginine containing protein can be citrullinated. Because of this, and as a consequence of the structural determinants of citrulline recognition by anti-citrullinated protein antibodies (ACPA) at the molecular level (5), ACPA display extensive cross-reactivity and frequently recognize various citrullinated proteins (6, 7). So far, this has hampered the identification of single antigens as triggers of this response, and it is likely that no such single antigen exists. From a clinical perspective, ACPA, together with rheumatoid factors, represent the most specific and clinically most relevant biomarker in this disease (8, 9). Despite strong clinical associations, the question whether or not these autoantibodies and/or the underlying citrullinated-protein-related T- and B-cell responses are involved in disease initiation and chronicity remains a matter of research and debate. However, the autoreactive B-cell immune response that generates ACPA has lately received much attention, especially because therapeutic depletion of CD20^+^ B cells has proven to be effective in established disease, in particular in the autoantibody-positive subset of patients (10–12). In addition, technology has advanced such that ACPA-expressing B cells can now be identified in, and isolated from peripheral blood and synovial fluid of affected patients, despite their very low frequency in the circulation (13–17). This allows for an in depth analysis of the BCR repertoire and of the molecular characteristics of the BCR. Besides, it allows to gain insight into the development of this disease-specific autoreactive B-cell response.

## Extensive V-domain *N*-glycosylation of ACPA-IgG

Previously, we could demonstrate that ACPA of the immunoglobulin (Ig)G isotype are extensively *N*-glycosylated in the V-domain (18). In fact, on average >90% of secreted ACPA-IgG molecules isolated from serum carry such glycans, which is in marked contrast to the much lower frequency of V-domain *N*-glycans observed in the total serum IgG repertoire. In-depth analysis of glycans found on ACPA-IgG V-domains revealed that these are expressed as fully-developed, biantennary *N*-glycans that frequently terminate with negatively charged sialic acid residues (19). This remarkable presence of glycans causes a shift in molecular size if affinity-purified ACPA-IgG or their corresponding F(ab)_2_-fragments are analyzed by gel electrophoresis or size-exclusion chromatography. As this is not the case for ACPA-IgM (20), we hypothesized that ACPA-IgG acquire *N*-glycans by introducing somatic mutations at positions that generate the *N*-glycosylation consensus sequence N-X-S/T (where X is any amino acid except proline), which is required for protein *N*-glycosylation. Such tripeptide sequons are only expressed by very few germline V-region genes. However, the repertoire of V-regions harbors multiple locations (termed progenitor sites) at which a single somatic mutation can lead to the generation of the sequon, i.e., of an *N*-glycosylation site (21). Indeed, recent sequence analysis of the V-regions of heavy chains (HC) of citrullinated antigen-specific BCRs showed a high frequency (>80%) of such *N*-glycosylation sites in the polyclonal ACPA-IgG repertoire, in contrast to a remarkable scarcity of such sites in a comparative, V-region matched repertoire of healthy donor IgG (22, 23). Notably, all of the *N*-glycosylation sites identified in the ACPA-IgG V-region repertoire were *de-novo* generated sites originating from somatic mutations, while no germline-encoded sites were observed (22).

## Low-Avidity and Cross-Reactivity of the ACPA Response

Interestingly, the B-cell response directed against citrullinated antigens generates a secreted polyclonal antibody repertoire that is of remarkably low-avidity (24). This low-avidity response can likely explain the concurrent presence of ACPA-IgM and ACPA-IgG, as IgM-expressing B cells will not be outcompeted by high-affinity IgGs normally seen during immune responses after e.g., tetanus vaccination. Nonetheless, the ACPA-response undergoes extensive isotype-switching (25) and somatic hypermutation (22), indicating that ACPA-expressing B cells are able to acquire T-cell help required for the expression of Activation Induced Cytidine Deaminase (AID). In addition, there is considerable cross-reactivity of the antibody pool toward different citrullinated proteins and toward other post-translational modifications (6, 26). On the clonal level, the response harbors clones that recognize citrullinated antigens only, while others are cross-reactive to antigens modified at lysine residues to express homocitrulline. However, all clones require the posttranslational protein modification as common determinant and do not bind the native protein. This cross-reactivity and the lack of high avidity are remarkable features, given that ACPA-IgG V-regions carry extensive somatic mutations. In fact, the number of nucleotide mutations in ACPA-IgG exceeds those of the total IgG repertoire and those of common, high-avidity vaccine responses directed against, for example, tetanus toxoid (22). Hence, ACPA-expressing B cells undergo extensive somatic mutation events, presumably during multiple cycles through GCs, yet their selection for survival does not seem to require an extensive increase of antibody affinity, as would be expected from a persistent immune response that is not clonally deleted (27).

## Non-Random Accumulation of *N*-glycosylation Sites

**Figure 1:**
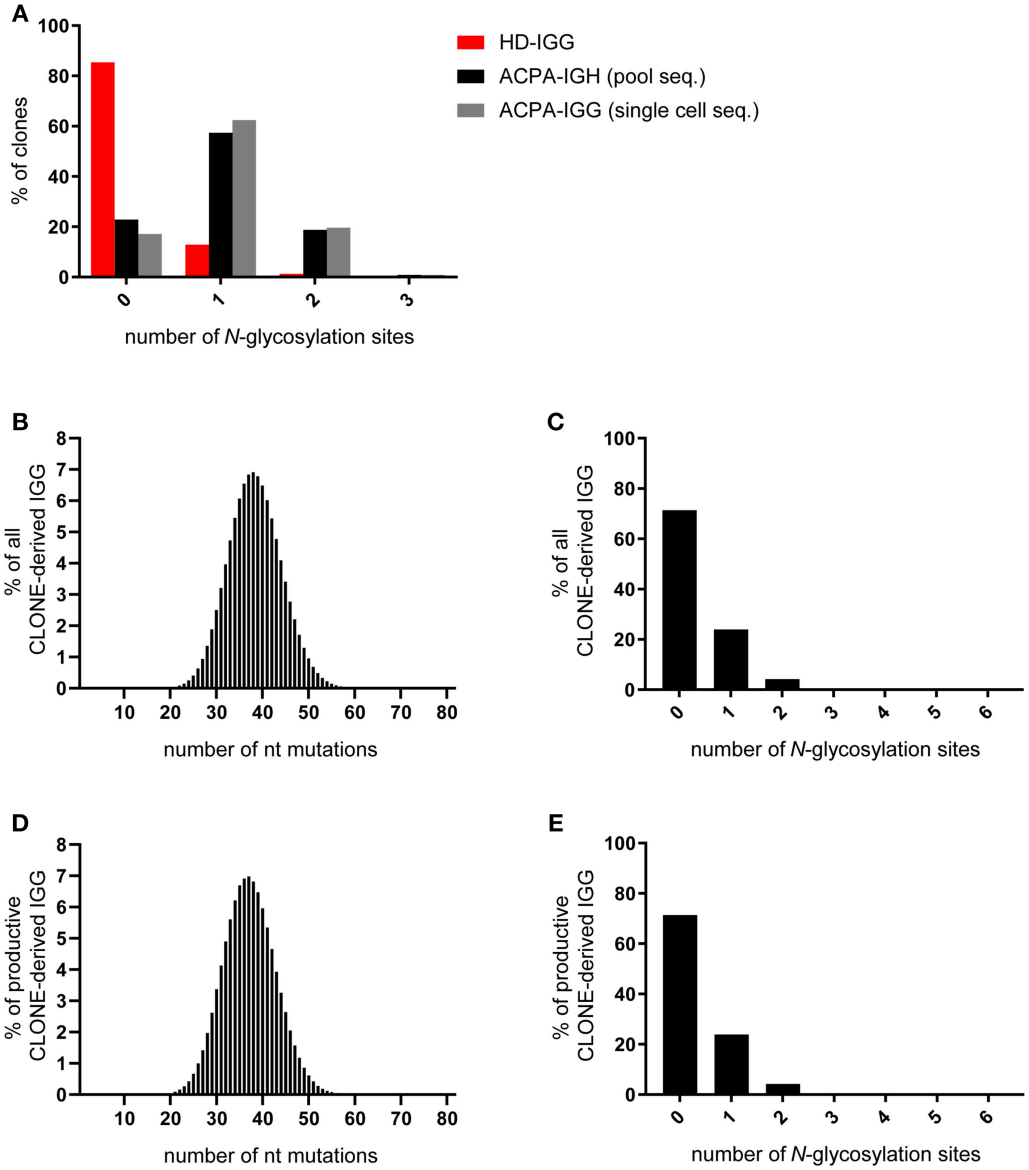
Frequency of *N*-glycosylation sites in the immunoglobulin heavy chain variable domain. **(A)** Percentage of ACPA- and healthy donor-derived IgG heavy chain clones containing zero, one, or more *N*-glycosylation sites in their BCR. Unique productive ACPA-derived clones were obtained using pool-sequencing (black bar) and single cell-sequencing (gray bar) and compared to unique productive pool-sequenced HD-IGG (red bar). **(B)** Frequency of nucleotide mutations in all sequences derived from the CLONE-algorithm with germline-reverted ACPA-IGG sequences as input data. **(C)** Percentage of all CLONE algorithm-derived clones containing zero, one, or more *N*-glycosylation sites. **(D,E)** Subset analysis exclusively on CLONE-algorithm derived productive sequences, without stop codons in IGHV, as analyzed in **(B,C)**.

Given the observations described, we hypothesized that ACPA-expressing B cells could be selected in GCs or GC-like structures based on the presence of *N*-glycosylation sites in the V-domain rather than on the affinity by which their cognate antigens are recognized. If correct, one could postulate that this alternative selection mechanism depends on the accumulation of *N*-glycosylation sites in the V-region, either at specific positions or dispersed over the V-region at high frequency with the presence of progenitor sites and/or the site-specific activity of AID [at so-called hotspot motifs (28)] governing their distribution. In fact, such an accumulation of *N*-glycosylation sites should not be a simple (“random”) reflection of frequent somatic mutations, but rather occur in both highly mutated clones and in those harboring fewer mutations. Indeed, our first analysis indicated that *N*-glycosylation sites in ACPA BCR IGHV-regions do not accumulate randomly over the V-region sequence. In fact, although we observed that somatic mutations were required for the generation of *N*-glycosylation sites, it was striking that the number of nucleotide mutations in ACPA IGHV-regions did not correlate with the number of *N*-glycosylation sites that were generated. Hence, zero, one or multiple *N*-glycosylation sites were observed in clones with few and frequent mutations, and not only highly mutated clones showed a high number of *N*-glycosylation sites (22). This latter finding is relevant as it suggests that the introduction of *N*-glycosylation sites is not the mere consequence of a high mutation rate. Rather, the mutation rate is likely a consequence of continuous (re-)activation of the autoreactive B-cell population, while the *N*-glycans could be involved in the provision of additional signals that provide a selective advantage to these cells. To further substantiate this notion, we performed a subsequent, in-depth analysis of *N*-glycosylation sites and their characteristics in the ACPA-IgG IGHV-region repertoire. Full length V-region sequences of IgG HC were obtained from single cell isolation of ACPA-expressing B cells using cyclic citrullinated peptide (CCP)2 as a model antigen. This model peptide has been designed for clinical diagnostic use to capture a broad repertoire of polyclonal ACPA. Importantly, antibodies recognizing this peptide are bona-fide ACPA that also recognize various citrullinated proteins (7). Cell isolation was followed by *A*nchoring *R*everse *T*ranscription of *I*mmunoglobulin *S*equences and *A*mplification by *N*ested PCR [“ARTISAN PCR” (29)] and Sanger sequencing. In addition, ACPA-expressing B cells were isolated as pools followed by ARTISAN-PCR and next generation sequencing (22). IgG sequences were selected for analysis from single-sorted ACPA-expressing B cells, whereas sequencing data from pool-sorted ACPA-expressing B cells contained heavy chain transcripts from IgG (~83%), IgA (~11%), and IgM (~6%) expressing cells. Sequences were analyzed using IMGT-(High)V-quest (30), ARGalaxy (31), and Microsoft Access to define BCR sequences as unique productive clones based on VJ rearrangement + CDR3(AA) sequence identity. In addition, we retrieved information about the introduction of *N*-glycosylation sites across the variable region. As described, we observed acquired, non-germline encoded, *N*-glycosylation sites in >80% of ACPA-IgG V-region genes. Our subsequent analysis revealed that most clones harbored one site per HC, while two or three sites per HC were also observed but less frequently (Figure 1A). Importantly, the frequency of sites was highly comparable between sequences of both isolation and sequencing techniques, excluding sequencing errors of either technique as a potential source of bias in the data. More specifically, 77% of pool-sorted ACPA B cells (223 clones) and 83% of single cell-sorted ACPA B cells (117 clones) contained one or more acquired sites (Figure 1A). In contrast, sites were absent in >80% of IgG-clones isolated from a healthy donor IgG HC repertoire. As expected, the number of mutations found in the healthy donor sequences was substantially lower compared to the mutation frequency observed in ACPA-IgG. In fact, a mean of almost 50 nucleotide mutations was observed in ACPA-IgG sequences compared to ~25 nucleotide mutations in the healthy donor repertoire (22). While this comparison demonstrates the remarkable degree of V-domain *N*-glycosylation of ACPA-IgG, we felt that caution should be taken as to a direct comparison of *N*-glycosylation site frequencies because of the substantially lower number of nucleotide mutations present in the healthy donor repertoire.

## Computational Germinal Center Simulation Supports the Concept of Selective Introduction of *N*-glycosylation Sites

To account for this difference in mutation frequency, we simulated the generation of *N*-glycosylation sites during the process of SHM in the GC response using an adapted, pre-existing computational model [CLONE (32, 33)] that provides a stochastic simulation of affinity maturation. The simulation is initiated with a single B cell associated to a selected germline sequence obtained from reverting IgG sequences (*n* = 163) obtained from ACPA-expressing B cells. This process necessarily conserved the CDR3 region. In the model, all cells divide once and acquire a Poisson distributed number of somatic mutations with an average that matches the number of mutations observed in the ACPA sequences as a proxy for repetitive cycles of B cell circulation through GCs. According to pre-defined probabilities, a mutation may have no effect, causes cell death (negative selection) or provides a selective advantage (positive selection) by decreasing its apoptosis rate. The simulation was terminated after 40 cell divisions. The simulation was repeated for each of the 163 input sequences and for each sequence we ran the simulation at least 10 times. The simulation yielded a Gaussian distribution of nucleotide mutations based on 26.799.339 clones (median of 37 nucleotide mutations per clone; Figure 1B). Despite this enhanced number of somatic mutations, the vast majority of clones did not harbor any *N*-glycosylation sites (Figure 1C). This was the case if all generated sequences were taken into account, but also if the large number of non-productive sequences that the algorithm generated with stop codons (81%) were removed from the analysis (Figures 1D,E). Together, these data indicate that the process of somatic hypermutation during GC-responses in the healthy situation, even if artificially “pushed” to mutation rates exceeding those of protective, antigen-specific responses, does not lead to the accumulation of *N*-glycosylation sites comparable to the degree observed for ACPA-IgG. This also indicates that *N*-glycans in the IGHV-domain do not contribute to the selection of (presumably) higher affinity clones, which is in line with the frequency of *N*-glycosylation sites in the healthy donor data set (Figure 1A), our observations from tetanus toxoid-specific clones (22), the low avidity of the ACPA response (24), and work from others (34). Hence, it is conceivable that additional, glycan-dependent selective mechanisms influence the accumulation of sites observed in the citrullinated antigen-directed, autoreactive B-cell response.

## *N*-glycosylation Sites Result From Single and Multiple Somatic Hypermutation Events

**Figure 2:**
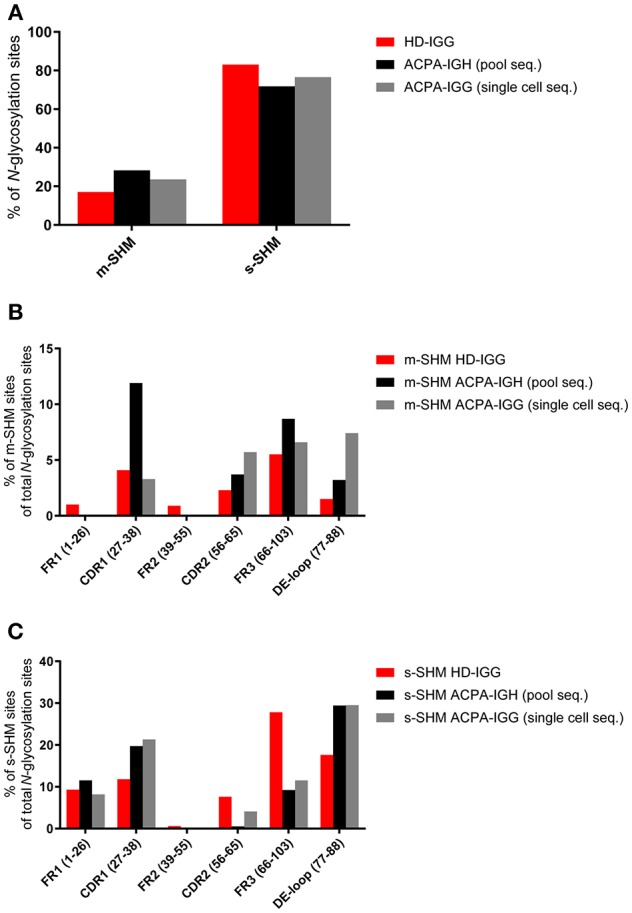
Frequency and distribution of m-SHM and s-SHM sites. Pool- (black bar) or single cell-sequenced (gray bar) ACPA clones and HD-IGG (red bar) clones were analyzed for the presence of *N*-glycosylation sites that require single or multiple somatic hypermutations (s-SHM or m-SHM). **(A)** Percentage of *N*-glycosylation sites that require single or multiple somatic hypermutations. **(B)** Distribution of m-SHM sites across the HC variable region. **(C)** Distribution of s-SHM sites across the HC variable region.

While only few germline-encoded immunoglobulin heavy chain variable (IGHV) region genes, such as IGHV1-8, IGHV4-34, and IGHV5-1, harbor an *N*-glycosylation site consensus sequence, there is an abundance of sequence positions which require only a single somatic mutation to generate a *de-novo N*-glycosylation site (so called progenitor glycosylation sites) (21). Additional sites can be formed on other positions but these require multiple mutations. Given the observations described above indicating the preferred outgrowth of ACPA-expressing B cells that have introduced *N*-glycosylation sites and a glycosylation-dependent selective mechanism, we hypothesized that single somatic hypermutation sites (s-SHM), i.e., sites generated from progenitor glycosylation sites, would be abundant in both the healthy donor and the ACPA-IgG repertoire. In contrast, ACPA-IgG V-regions would be expected not only to harbor a higher number of sites originating from multiple mutations (m-SHM) in absolute terms, but also in relative terms when corrected for the total number of *N*-glycosylation sites in both data sets. Indeed, while s-SHM sites were present in both sets of data, ACPA-IgG harbored ~9% more m-SHM sites. Again, this finding was observed irrespective of the sequencing approach (Figure 2A). Of note, within the ACPA repertoire, certain regions within the IGHV-domain, such as complementarity determining region (CDR) 2 and framework region 2 (FR2) more frequently harbored m-SHM sites, whereas s-SHM sites were most abundant in FR1 (Figures 2B,C). Besides, *N*-glycosylation sites were more frequent in the DE-loop [the loop between the second and third CDR, position 77–88 of FR3 (21)] than in the remaining part of FR3. The *N*-glycosylation sites in the remaining part of FR3 required more often multiple mutations compared to sites in the DE-loop. Possibly, the distribution of *N-*glycosylation sites is also influenced by the skewed activity of AID toward hotspot motifs. Indeed, a random analysis of a set of *N*-glycosylation sites and progenitor sites indicated that some but not all acquired sites were situated on AID hotspot motifs (WRCY/WA, data not shown). In addition, Koers et al. (35) analyzed the relative occurrence of “other” mutations on the progenitor sites than the *N*-glycosylation site inducing mutations. Overall, this analysis indicated that there was no intrinsic bias concerning the introduction of *N*-glycosylation sites. In summary, these data show that ACPA-IgG contain not only an absolute and relative increase in *N*-glycosylation-sites, but also indicate that these *N*-glycosylation sites are more often acquired by multiple mutations. Again, these data are in line with the notion that the introduction of *N*-linked glycans in the V-domain of ACPA confers an advantage to citrullinated-protein directed B cells.

## Physicochemical Properties of *N*-glycosylation Sites in ACPA-IgG HC

**Figure 3:**
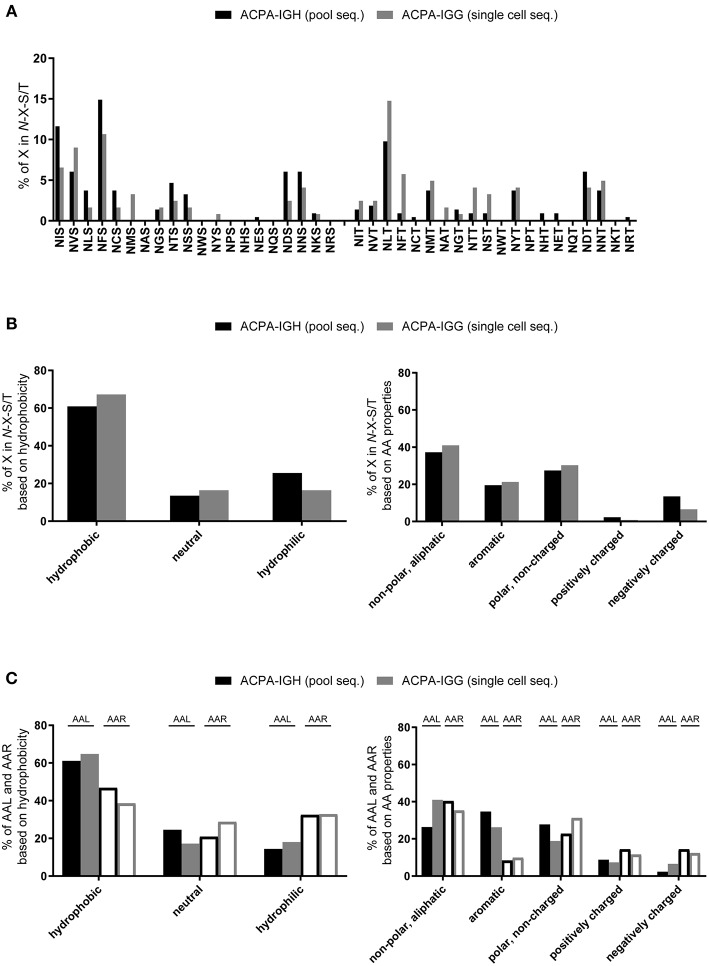
In depth analysis of amino acid “X” in *N*-glycosylation sites. Pool- (black bar) and single cell-sequenced (gray bar) ACPA clones were analyzed for the properties of amino acid “X” in the consensus sequence N-X-S/T. **(A)** Frequency of all amino acids used at position “X” in *N*-glycosylation sites. **(B)** Analysis of the hydrophobicity of the amino acids on position “X” and of their non-polar (aliphatic), aromatic, polar (non-charged), positively-, and negatively-charged characteristics. **(C)** Amino acids left (AAL, closed bar) and right (AAR, open bar) flanking *N*-glycosylation sites ordered by hydrophobicity and by their non-polar (aliphatic), aromatic, polar (non-charged), positively-, and negatively-charged characteristics.

As the mechanism by which V-domain *N*-glycans influence the selection and survival of ACPA-expressing B cells is unclear, we determined the molecular characteristics of the *N*-glycosylation tripeptide sequons with the aim of defining features relevant to such sites in ACPA-IgG. Specifically, we determined the physicochemical properties of the amino acid “X” in “N-X-S/T” and analyzed its charge and hydrophobicity (Figure 3). These analyses revealed that most amino acids at position “X” (except, by definition, proline) could participate in the formation of *N*-glycosylation sites (Figure 3A), with a certain preference for hydrophobic, non-charged amino acids (Figure 3B). In general, no specific pattern or choice of particular amino acids was observed either at position “X” or at the flanking residues (Figure 3C), indicating that the presence of the *N*-glycan itself rather than the structural properties of the amino acid backbone are relevant in the aberrant selection of ACPA-expressing B cells. This observation is important, as the presence of an *N*-glycosylation site in a given protein does not necessarily imply the presence of a glycan. In fact, localization of the site and the conformational folding of the protein might preclude the use of the site for glycosylation, while it could still have functional relevance for the antibody as such. Nonetheless, analysis of 27 recombinantly produced monoclonal antibodies with, in total, 38 sSHM *N*-glycosylation sites in different positions suggests that a high frequency of these sites, and probably other sSHM sites, is occupied by *N-*glycans (35).

## Clinical Relevance of ACPA-IgG V-domain *N*-glycosylation

As described, V-domain *N***-**glycosylation of ACPA-IgG provides insight in factors that are relevant to the development and selection of citrullinated antigen-directed B cells. However, the presence of this feature presumably also reflects the help from T cells to ACPA-expressing B cells, and as such has implications for its use as a clinical biomarker. Serum ACPA-IgG can persist for many years in the absence of disease-specific symptoms (36, 37). In fact, not all ACPA-positive individuals will develop RA (38–41). Hence, it is of clinical relevance to identify those individuals who transition from symptom-free autoimmunity to clinically overt autoimmune disease. In this context, it is intriguing that the strongest genetic risk factor for ACPA-positive RA, the HLA shared epitope alleles, confer risk only to ACPA-positive disease but not to ACPA-positivity, i.e., to autoimmunity without clinical symptoms (42, 43). As HLA-molecules are crucial for the communication between T and B cells, it is possible that the transition from autoimmunity to overt, ACPA-positive disease is triggered by T cells that provide help to ACPA-expressing B cells. In doing so, this T cell help is likely to induce or enhance somatic mutations and, hence, the generation of *N*-glycosylation sites. Indeed, first evidence obtained from the study of ACPA-IgG in asymptomatic ACPA-positive individuals indicates that the degree of serum ACPA-IgG V-domain *N***-**glycosylation is markedly lower than in RA patients (44). Moreover, although analyzed so far in only a small cohort of patients, the presence of extensive V-domain *N***-**glycosylation in serum ACPA-IgG predicted RA-development. Together, these observations demonstrate that the generation of V-domain glycans and the biological consequences might be directly associated to disease-relevant processes, either caused by the activation of disease-relevant T cells, the outgrowth of the autoreactive B-cell population itself or by the secreted autoantibody repertoire. The exact biological mechanism by which the presence of V-domain glycans on ACPA-IgG influence the selection and outgrowth of autoreactive B cells is, however, still unknown. Potential effects mediated by the glycans include the modulation of antigen binding and/or of BCR signaling, or the direct binding to lectins expressed on either the same or neighboring cells (45). Although we did not observe an overrepresentation of *N*-glycosylation sites in the CDR regions, which are supposed to be the most important regions for antigen binding, subtle changes in antigen binding between glycosylated and non-glycosylated monoclonal ACPAs have already been described (18). Hence, additional, in-depth investigations are required to further dissect the biological effector functions associated with these ACPA-IgG V-domain glycans.

## Summary and Conclusion

Taken together, we here describe the detailed analysis of the occurrence of *N*-glycosylation sites in the V-domain of a human autoreactive B-cell response and discuss its potential relevance for autoreactive B-cell development. Our data indicate that the extensive *N*-glycosylation found in ACPA-IgG is not the result of the random accumulation of *N*-glycosylation sites due to extensive SHM. In fact, we propose the concept that the *N*-glycosylation is involved, either directly or indirectly, in the (positive) selection of autoreactive B-cell clones, even in the absence of a high affinity recognition of the self-antigen. However, as discussed above, the functional mechanisms by which V-domain *N*-glycans promote positive selection (or inhibit negative selection) are incompletely understood. As different B-cell responses have been described that demonstrate enhanced levels of V-domain *N*-glycans and/or glycosylation sites, such as those observed in Sjögren's syndrome (46), those found in follicular lymphoma (47–49), those generating anti-hinge region antibodies, and those in immunoglobulin subclass G4-related disease and primary sclerosing cholangitis (50), it is likely that the mechanisms operational in the development of ACPA-expressing B cells are not restricted to this B-cell response only. So far, however, we have not identified another antigen-specific B-cell response that generates a degree of V-domain *N*-glycosylation comparable to that of ACPA-IgG. In conclusion, future studies will need to profit from this extreme phenotype and focus on the consequences of V-domain *N*-glycans for the recognition and binding strength of citrullinated antigens, on modulation of B-cell receptor-mediated signaling events, and on potential binding interactions between V-domain *N*-glycans and lectins.

## Ethics Statement

This study was approved by the Institutional Review Board of Leiden University Medical Center. All subjects involved in the study gave written informed consent prior to any study-related procedure.

## Author Contributions

RV, LS, BS, and MK designed the study, performed the experiments, and analyzed the data. TR contributed to the design of the study, provided algorithms for data analysis, interpreted the data, and critically revised the manuscript. AK, RT, and HS designed the study, analyzed data, and wrote the manuscript.

### Conflict of Interest Statement

RT and HS are co-authors on a provisional patent application based on research related to topics discussed in this study. The remaining authors declare that the research was conducted in the absence of any commercial or financial relationships that could be construed as a potential conflict of interest.
